# Carbomer Hydrogels with Microencapsulated α-Tocopherol: Focus on the Biocompatibility of the Microcapsules, Topical Application Attributes, and In Vitro Release Study

**DOI:** 10.3390/pharmaceutics16050628

**Published:** 2024-05-07

**Authors:** Ljiljana Đekić, Jelena Milinković Budinčić, Dušanka Stanić, Jadranka Fraj, Lidija Petrović

**Affiliations:** 1 Department of Pharmaceutical Technology and Cosmetology, Faculty of Pharmacy, University of Belgrade, 11221 Belgrade, Serbia; 2 Department of Pharmaceutical Engineering, Faculty of Technology Novi Sad, University of Novi Sad, 21000 Novi Sad, Serbia; jelenamilinkovic@uns.ac.rs (J.M.B.); jadranka@uns.ac.rs (J.F.); lidijap@uns.ac.rs (L.P.); 3 Department of Physiology, Faculty of Pharmacy, University of Belgrade, 11221 Belgrade, Serbia; dusanka.stanic@pharmacy.bg.ac.rs

**Keywords:** α-tocopherol, microencapsulation, complex coacervation, formaldehyde, glutaraldehyde, chitosan, sodium lauryl ether sulphate, carbomer hydrogel, stability, in vitro release

## Abstract

The microencapsulation of α-tocopherol based on the complex coacervation of low-molecular-weight chitosan (LMWC) and sodium lauryl ether sulphate (SLES) without harmful crosslinkers can provide biocompatible carriers that protect it from photodegradation and air oxidation. In this study, the influence of the microcapsule wall composition on carrier performance, compatibility with a high-water-content vehicle for topical application, and release of α-tocopherol were investigated. Although the absence of aldehyde crosslinkers decreased the encapsulation efficiency of α-tocopherol (~70%), the variation in the LMWC/SLES mass ratio (2:1 or 1:1) had no significant effect on the moisture content and microcapsule size. The prepared microcapsule-loaded carbomer hydrogels were soft semisolids with pseudoplastic flow behavior. The integrity of microcapsules embedded in the hydrogel was confirmed by light microscopy. The microcapsules reduced the pH, apparent viscosity, and hysteresis area of the hydrogels, while increasing their spreading ability on a flat inert surface and dispersion rate in artificial sweat. The in vitro release of α-tocopherol from crosslinker-free microcapsule-loaded hydrogels was diffusion-controlled. The release profile was influenced by the LMWC/SLES mass ratio, apparent viscosity, type of synthetic membrane, and acceptor medium composition. Better data quality for the model-independent analysis was achieved when a cellulose nitrate membrane and ethyl alcohol 60% *w/w* as acceptor medium were used.

## 1. Introduction

Vitamin E is commonly used as an umbrella term for a group of chemical isomers including α-, β-, γ-, and δ-tocopherols and tocotrienols that share a hydrophilic chromanol ring, which provides high antioxidant activity, and a hydrophobic 16-carbon phytyl side chain. The side chain of tocopherols is saturated, whereas, in tocotrienols, it has three conjugated double bonds. There are four isomers within both subgroups, which differ in the number and position of methyl groups on the chromanol ring [1]. Vitamin E is often used as a stabilizer in food, pharmaceuticals, and cosmetic products, protecting the ingredients from oxidation and preventing the spoilage of the products. Although the biological effects of vitamin E are the subject of ongoing scientific debate, it has been used as a dietary supplement and as an ingredient with therapeutic or cosmetic effects in pharmaceuticals and cosmetic products, respectively [2,3,4,5,6]. The therapeutic and cosmetic effects of vitamin E on the skin have been the subject of numerous scientific studies in recent decades. The topical application of vitamin E is thought to improve wound healing and reduce scarring, and it may be beneficial in the treatment of skin diseases such as *granuloma annulare*, melanoma, and other cancers [7,8,9,10,11,12]. Vitamin E in the form of α-tocopherol acetate is an essential skin moisturizer in hand sanitizer gels with a high alcohol content (up to 70% isopropyl alcohol or ethyl alcohol), whose daily use has been widespread worldwide since the beginning of the COVID-19 pandemic and continues even after the pandemic. In skin-care products, vitamin E keeps the skin in good condition by increasing the moisturizing capacity of the epidermis due to occlusion and prevents premature skin aging by protecting cell membranes, lipoproteins, and depot fat from oxidation caused by UV radiation [13,14,15,16]. The European regulations for cosmetic products allow the use of all four tocopherol isomers under the single INCI (International Nomenclature Cosmetic Ingredient) name *tocopherol* [17], with α-tocopherol being the most biologically active [18]. Furthermore, recent initiatives emphasize that only α-tocopherol can be considered a biomedically relevant isomer [19]. α-Tocopherol (IUPAC name: (2*R*)-2,5,7,8-tetramethyl-2-[(4*R*,8*R*)-4,8,12-trimethyltridecyl]-3,4-dihydrochromen-6-ol)) is an odorless, clear light yellow viscous liquid that oxidizes and darkens when exposed to air or light [20]. It is insoluble in water, but freely soluble in ethanol [21] and miscible with vegetable oils [22,23]. It is very susceptible to chemical degradation when exposed to heat, light, oxygen, transition metal ions, and alkaline conditions, and it can be absorbed into plastic [5]. Its low water solubility, chemical instability, and potential incompatibility with plastic containers make the development of topical formulations containing α-tocopherol a major challenge. The inherent disadvantages of the α-tocopherol molecule may also affect the later stages of the product’s life cycle due to the lower active ingredient content and the risk of the formation of potentially toxic chemical degradation products, including direct photolysis, when the product is exposed to daylight [24]. Therefore, despite the recognized benefits of α-tocopherol as a component of topical formulations, poor water-solubility and high chemical instability are permanent obstacles that need to be overcome in order to develop a stable and effective product.

The encapsulation of α-tocopherol in nanoscale (1–1000 nm) and microscale (1–1000 μm) carriers is a widely explored approach to overcome its chemical instability and enable its incorporation into aqueous vehicles and/or controlled delivery from the product [25,26,27]. A comprehensive review article by Ribeiro et al. [28] highlights the advantages of microencapsulation of α-tocopherol in overcoming low solubility, poor shelf life, or loss of activity. Microencapsulation technologies that focus on the use of biocompatible and environmentally friendly materials, that ensure high encapsulation efficiency, and are feasible on an industrial scale are preferable [29,30]. The encapsulation of α-tocopherol in microcapsules with an oily core surrounded by a polymer wall is a relatively simple and cost-effective approach to protect the microcapsule core from factors affecting its stability and to convert the lipophilic liquid into a powder suitable for homogeneous distribution in vehicles with higher polarity [31,32]. Microcapsule wall properties, including the biocompatibility, mechanical characteristics, ability to moisten and swell in an aqueous environment, and active substance-release control mechanisms, are directly related to the wall material and microencapsulation technique. Therefore, the selection of microcapsule wall components and the determination of wall properties are important initial aspects of product development that need to be addressed. The use of natural biocompatible and biodegradable polymers is a current focus in regard to the microencapsulation of active pharmaceutical and cosmetic ingredients [33,34]. Chitosan is a linear biopolyaminosaccharide obtained by partial or complete N-deacetylation of chitin (poly β-(1–4)-N-acetyl-D-glucosamine)-originating renewable sources such as the exoskeletons of crustaceans, insects, and fungal cell walls [35]. Chitosan has three types of reactive groups in its structure: an amino group (-NH2) at position S-2, and primary and secondary hydroxyl groups (-OH) at positions S-3 and S-6 [36]. It is soluble in water at pH < 6.5 when it has a polycationic character that allows for interactions with oppositely charged surfactants and polymers to form coacervates that can be adsorbed at the interface of oil-in-water emulsions and form a microcapsule wall around the oil core [35,37,38,39]. This phenomenon is known as *complex coacervation* and is an effective method for the microencapsulation of lipophilic active compounds due to the high payload, high encapsulation efficiency, and mild processing conditions [40,41]. Since the coacervate is formed by ionic bonds, additional chemical crosslinking with formaldehyde or glutaraldehyde is a common strategy to strengthen the wall [42]. Although crosslinking involves covalently bonding aldehyde groups to amino groups of chitosan, it is important to be aware that formaldehyde and glutaraldehyde are known skin sensitizers [43]. In addition, formaldehyde has been classified as carcinogen and skin sensitizer according to European Commission Regulation No. 1223/2009 on cosmetic products, and as such is listed in Annex II of prohibited substances in cosmetic products [44]. Therefore, there is a clear interest in development of biocompatible chitosan-based microcapsules that are free of crosslinkers and suitable for topical administration. In the previously published study by our group [45], it was shown that microcapsules loaded with α-tocopherol can be successfully prepared both with and without aldehyde crosslinkers by the spray drying of an oil-in-water emulsion using the complex coacervation of low molecular weight chitosan (LMWC) and sodium lauryl ether sulfate (SLES). The method is suitable for producing spherical microcapsules in the form of powder with low moisture content (up to 1.76%), high encapsulation efficiency (>70%), and good dispersibility in water. When comparing α-tocopherol release, the LMWC/SLES microcapsule wall without crosslinking agent was found to allow the diffusion of the lipophilic active in a more controlled manner that those containing formaldehyde or glutaraldehyde. Future topical application of microencapsulated α-tocopherol requires incorporation into a suitable vehicle, and the compatibility of the microcapsules and the vehicle as well as the release of α-tocopherol from the formulation are crucial prerequisites for the performance of the formulation [46]. Research data on the behavior of encapsulated α-tocopherol in topical formulations are very limited [47]. Therefore, in this study, the investigation was continued with the aim of testing the suitability of the α-tocopherol-loaded LMWC/SLES microcapsules for the formulation of topical hydrogels. 

Topical hydrogels are semisolids consisting of an organic macromolecule, which is a gelling agent forming a coherent three-dimensional network and a liquid phase immobilized therein [48,49]. The liquid phase of topical hydrogels consists of purified water or mixtures of water and ethyl alcohol or isopropyl alcohol with the addition of humectants, such as glycerin or propylene glycol (10–20%), which also serve as film plasticizers after application [50,51,52]. The most commonly used gelling agents are high molecular weight polymers of acrylic acid crosslinked with allyl ethers of polyalcohols, known under the generic name of *carbomers* [53], as they form topical hydrogels with a number of advantages: they are easy to apply to intact and damaged skin and can be easily washed off the application site, they spread well, form a thin film that moisturizes the skin and cools it due to the rapid evaporation of the liquid phase, and prolong the residence time of the active ingredient at the application site. Carbomer Carbopol^®^ Ultrez 10 is a gelling agent of particular interest due to its environmentally friendly synthesis, its suitability for forming clear gels with water or water/alcohol mixtures, and its pronounced ability to be wetted by the aqueous phase and to disperse rapidly at relatively low stirring speeds which facilitates the preparation of hydrogels without lump formation [54,55]. Carbomer hydrogels with microencapsulated active ingredients potentially combine the advantages of microencapsulation and the hydrogel vehicle. However, the risk of compromising the stability of the carbomer hydrogel and the suitability for topical application after incorporation of microcapsules should be carefully evaluated. As far as we are aware, the formulation of carbomer hydrogels with microencapsulated α-tocopherol has not been reported anywhere. In addition, as mentioned, our previous results [45,56] have shed light on the possibility of avoiding aldehyde crosslinkers in the LMWC/SLES microcapsule wall. However, the importance of the crosslinker as well as the mass ratio of LMWC and SLES in the crosslinker-free microcapsule wall for the formulation of topical hydrogels remained unexplored. The aim of this study was to formulate carbomer hydrogels with microencapsulated α-tocopherol and to investigate the influence of the type and concentration of crosslinker and the mass ratio of LMWC/SLES in a crosslinker-free microcapsule wall on their compatibility with the hydrogel and suitability for topical application. Additionally, the in vitro α-tocopherol release in hydrogels with crosslinker-free microcapsules was evaluated.

## 2. Materials and Methods

### 2.1. Materials

The components of the microcapsule wall were low-molecular-weight chitosan (LMWC) (50,000–190,000 g/mol) (Sigma-Aldrich, Beijing, China) and sodium lauryl ether sulphate (SLES) with 2–3 ethylene oxide groups (Clariant, München, Germany). The degree of deacetylation of LMWC was determined to be 81.8% using the potentiometric titration method developed by Yuan et al. [57]. SLES was in the form of a sticky paste containing 70% of the active ingredient. A 36.5% formaldehyde solution (Sineks Medical, Belgrade, Serbia) or a 50% glutaraldehyde solution (Fisher Chemical*,* Loughborough, UK) was used to prepare the microcapsules containing a crosslinking agent. The oily core of the microcapsules consisted of caprylic/capric triglyceride (Saboderm TCC, Sabo S.p.A, Levate, Italy) and (±)-α-tocopherol (Sigma-Aldrich, Merck, Darmstadt, Germany). The purity of α-tocopherol was 96%. The following reagents were also used to prepare the microcapsule: acetic acid (Zorka-Pharma, Šabac, Serbia), sodium acetate (Centrohem, Stara Pazova, Serbia), silicon dioxide (Aerosil 200 Pharma, Evonik, Hanau, Germany), and purified water. 

The following substances were used to produce blank hydrogel: carbomer (Carbopol^®^ Ultrez 10 polymer, Lubrizol, Wickliffe, OH, USA), triethanolamine (Sigma-Aldrich, Merck, Darmstadt, Germany), isopropyl alcohol (Fagron, Capelle aan den Ijssel, The Netherlands), propylene glycol (Fagron, Capelle aan den Ijssel, The Netherlands), and purified water. 

The ingredients of artificial sweat (sodium chloride (NaCl), calcium chloride (CaCl_2_), magnesium sulfate (MgSO_4_), potassium dihydrogen phosphate (KH_2_PO_4_), and sodium hydroxide (NaOH)) were purchased from Sigma-Aldrich, Merck (Darmstadt, Germany). 

In the preparation of the acceptor media for the in vitro release tests were used: ethyl alcohol 96% *v/v* (Zorka-Pharma, Šabac, Serbia), polysorbate 20 (Fagron, Capelle aan den Ijssel, The Netherlands) and purified water.

### 2.2. Methods

#### 2.2.1. Preparation of Microcapsules with α-Tocopherol

**Figure 1:**
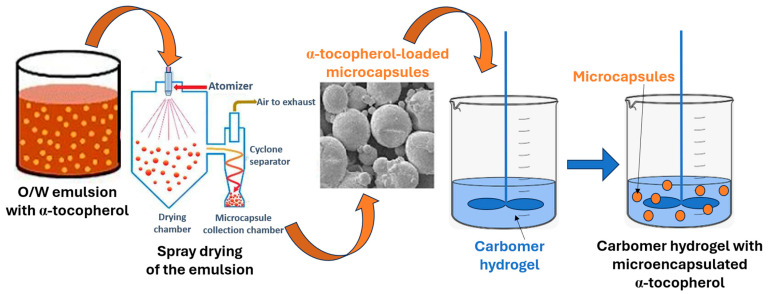
Schematic representation of microencapsulation of α-tocopherol by spray drying oil-in-water (O/W) emulsion and subsequent preparation of a hydrogel with the microencapsulated α-tocopherol by dispersing the microcapsules in the freshly prepared carbomer hydrogel.

α-Tocopherol-loaded microcapsules with and without aldehyde crosslinkers were prepared using the complex coacervation and spray drying method described in our previous work [45]. In brief, stock solutions of LMWC 0.2% *w/w* and SLES 0.4% *w/w* were prepared and in both the pH was adjusted and maintained at pH 4.0 (827 lab pH-meter, Metrohm, Herisau, Switzerland) with acetate buffer. The required volumes of stock solutions were mixed to achieve a LMWC/SLES mass ratio 2:1. After 24 h, these mixtures were used to stabilize oil-in-water emulsions prepared with a mass ratio of 20:80, the oil phase being a solution of α-tocopherol (10% *w/w*) in oxidation-resistant caprylic/capric triglycerides [56]. These emulsions were prepared by stepwise addition of the oil phase into the aqueous phase during the first minute of homogenization and homogenization was continued with the Ultra Turrax T25 (IKA-Werke GmbH & Co. KG, Staufen, Germany) at 5000 rpm and 30 °C for 9 min. For the purpose of separating oil droplets, Aerosil was added in a concentration of 2% *w/w*. To prepare the microcapsules with crosslinked wall, the crosslinker (formaldehyde or glutaraldehyde) was added in a mass ratio of LMWC/crosslinker of 1:1 and 1:2 to the emulsions. The emulsions were left on a magnetic stirrer for 2 h with slow stirring and then spray dried in a Büchi Mini Spray Dryer B-190 (Büchi AG, Flawil, Switzerland) with a standard nozzle of 0.7 mm, to obtain microcapsules (Figure 1). The controlled parameters of spray drying were aspiration (0.6 m^3^/min), feeding (2.2 mL/min), inlet temperature (160 °C) and outlet temperature (100 °C). The crosslinker-free microcapsules were prepared using the same procedure without the crosslinkers. Two types of α-tocopherol-loaded crosslinker-free microcapsules were prepared using the LMWC/SLES coacervates at mass ratios of 2:1 and 1:1. 

#### 2.2.2. Characterization of α-Tocopherol-Loaded Microcapsules

α-Tocopherol-loaded microcapsules with crosslinkers and crosslinker-free microcapsules based on LMWC/SLES coacervate in a mass ratio of 2:1 were characterized in terms of moisture content, microparticle mean diameter, and encapsulation efficiency (EE) in the previous work [45]. To continue the research, the de novo prepared crosslinker-free microcapsules based on LMWC/SLES coacervate in a 1:1 mass ratio were characterized using the same methodology. In brief, the moisture content was determined gravimetrically by oven-drying 1 g of microcapsule powder at 105 °C to constant weight. The experiment was carried out in triplicate. The size distribution of the microcapsules in the aqueous suspension was determined by analyzing of photomicrographs taken with a light microscope Biooptica BEL-3000 (Resolab GmbH, Bad Oeynhausen, Germany) at 40× magnification, using BELView software (https://belview.software.informer.com/, accessed on 2 May 2024). The mean diameter of the microparticles, expressed as volume–surface area mean value d_vs_ (μm) and the standard deviation σ (μm) were calculated using the following equations:

d_vs_ = ∑n_i_ d_i_^3^/∑n_i_d_i_^2^ (1)

σ = (∑n_i_(d_i_ − d_vs_)^2^/∑n_i_)^1/2^ (2)

where d_i_ is the microcapsule diameter (μm), and n_i_ is the number of microcapsules. The EE was determined by extracting the encapsulated α-tocopherol from a sample of 0.1 g microcapsules with 80% *w/w* ethyl alcohol after 5 min of vigorous stirring in a Vortex-Genie 2 mixer (Scientific Industries Inc., New York, NY, USA). The sample was then filtered (Sartorius filter, 0.1 μm size) and the content of extracted α-tocopherol was determined spectrophotometrically (Halo DB-20S UV–vis spectrophotometer, Dynamica Ltd., Mablethorpe, UK) at 290 nm and calculated from the linear analytical curve:

y = 69.586x − 0.0163

corresponding to the α-tocopherol concentration range of 0.001–0.006% and a correlation coefficient of 0.9985. The EE was calculated according to Equation (3):

E = m_m_/m_t_ · 100% (3)

where m_m_ is the mass of α-tocopherol extracted from 1 g of microcapsules, and m_t_ is the mass of α-tocopherol added during emulsification of the oil phase. The experiment was carried out in triplicate.

#### 2.2.3. Preparation of Hydrogels with Microencapsulated α-Tocopherol

The hydrogels with microencapsulated α-tocopherol were prepared by dispersing the microcapsules in the previously prepared carbomer hydrogel (Figure 1). 

The blank carbomer hydrogel consisted of carbomer (0.5% *w/w*), triethanolamine (0.5% *w/w*), propylene glycol (10% *w/w*), isopropyl alcohol (25% *w/w*), and purified water (up to 100%). The purified water was freshly boiled and cooled to room temperature, immediately before the preparation of the blank hydrogel, to reduce the risk of microbial growth. The blank hydrogel was prepared by gradually dispersing carbomer powder in purified water while stirring with the IKA RW 20 digital propeller laboratory mixer (IKA^®^-Werke GmbH & Co. KG, Staufen, Germany) at a speed of 300 rpm. Propylene glycol and isopropyl alcohol were added to the carbomer dispersion, while stirring at the same speed. The stirring speed was then increased to 400 rpm, and neutralization was carried out by adding triethanolamine in the form of a 10% aqueous solution. 

Each hydrogel containing microencapsulated α-tocopherol was prepared by dispersing the required quantity of microcapsules in a measured amount of blank carbomer hydrogel, stirring at a speed of 300 rpm until a homogeneous semisolid was obtained. The concentration of microcapsules in each hydrogel was 5% *w/w*, while the remaining 95% was the hydrogel vehicle. The hydrogels were filled into aluminum tubes immediately after their preparation to protect them from light and evaporation during storage. 

#### 2.2.4. Evaluation of the Compatibility of Microencapsulated α-Tocopherol with Carbomer Hydrogel 

The prepared carbomer hydrogel without microcapsules (blank hydrogel) and the hydrogels with microencapsulated α-tocopherol were tested for appearance (color, consistency, and homogeneity), odor, pH, and rheological behavior 48 h after preparation. Color, homogeneity, and consistency were assessed visually. The pH value was measured using the potentiometric method with a pH-meter HI 9321 (Hanna Instruments Inc., Woonsocket, RI, USA), under ambient conditions, at 20 ± 3 °C. Flow behavior and apparent viscosity, as parameters of rheological behavior, were determined using a Physica Rheolab MC 120 rheometer/viscosimeter (Anton Paar, Ostfildern, Germany) equipped with an MK22 cone-plate measuring system. The measurements were performed at a temperature of 20 ± 0.1 °C, by subjecting the hydrogel sample to a continuous increase in shear rate from 0 to 200 s^−1^ and then a continuous decrease from 200 to 0 s^−1^. The collected data were used to create ascending and descending sequences of the flow curves. 

To verify the long-term compatibility of the microcapsules with the carbomer hydrogel, the evaluation appearance, pH measurement, and characterization of rheological behavior were repeated as described after 1 and 2 months of storage at controlled room temperature (20–25 °C), in accordance with the packaging and storage requirements of USP 43-NF 38 Chapter <659> [58]. In addition, the hydrogels with microencapsulated α-tocopherol stored for 2 months were examined by light microscopy using Olympus BX51P + cellSens Entry Version 1.14 (Olympus, Tokyo, Japan).

#### 2.2.5. Evaluation of Topical Application Attributes of the Hydrogels with Microencapsulated α-Tocopherol

In order to compare the influence of the crosslinking agent or its absence on the topical application attributes of the hydrogels with microencapsulated α-tocopherol, the samples of the hydrogels were subjected to a characterization of the properties relevant for skin application such as spreadability and dispersibility in artificial sweat, 48 h after preparation. 

The spreadability of the hydrogels on an inert flat surface was evaluated by placing a precisely measured sample (100 mg) of the hydrogel in the center of a Petri dish with a diameter of 9 cm. Immediately afterwards, the sample was covered with a glass plate of the appropriate diameter and weighed down with a 50 g weight. After the sample had been exposed to the load for 5 min, its diameter was measured horizontally and at an angle of 90° to the horizontal, and the mean diameter was calculated. The mean diameter was determined in triplicate for each hydrogel, and the spreadability diameter Φ (mm) was calculated as the mean of three mean diameter values with the corresponding standard deviation.

The suitability of the hydrogels with microencapsulated α-tocopherol for dispersion in artificial sweat was evaluated according to the method described by Djekic et al. [59]. Artificial sweat was prepared according to Shimamura et al. [60], by dissolving sodium chloride (49.96 mM), calcium chloride (0.15 mM), magnesium sulfate (1.00 mM), and potassium dihydrogen phosphate (7.50 mM) in purified water. The pH of the solution was adjusted to 5.4 with 0.1 M sodium hydroxide solution. The hydrogel sample (1.0 g) was measured in an Erlenmeyer flask, 9.0 g of freshly prepared artificial sweat was added, and the mixture was shaken on the laboratory shaker IKA KS 260-Basic (IKA^®^-Werke GmbH & Co. KG, Staufen, Germany) at a speed of 300 rpm. During shaking, the appearance of the dispersion was observed and recorded after 1 min, 2 min, and 5 min. 

#### 2.2.6. In Vitro Release Testing of the Hydrogels with Microencapsulated α-Tocopherol

**Figure 2:**
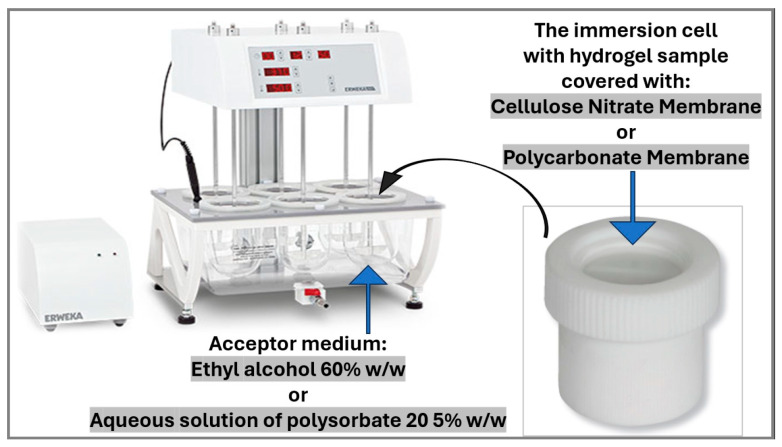
Schematic representation of the experimental design of the in vitro release test, including investigated variables: the membrane (cellulose nitrate membrane or polycarbonate membrane) and the acceptor medium (ethyl alcohol 60% *w/w* or aqueous solution of polysorbate 20 5% *w/w*).

For hydrogels with crosslinker-free microcapsules, the release kinetics of α-tocopherol were investigated according to USP 43-NF 38 Chapter <1724> Semisolid Drug Products—Performance Tests [61], using an immersion cell (VanKel Technology Group Inc., Raleigh, NC, USA) and a USP II apparatus with a rotating paddle Erweka DT 70 (Erweka, Langen (Hessen), Germany). A hydrogel sample of 2.0 g was filled into the immersion cell and covered with a membrane. Two types of membranes were used for the test: a cellulose nitrate membrane with a pore size of 0.45 µm (Cellulose Nitrate Membrane Filters, Whatman, Merck Life Science UK Limited, Gillingham, UK) and a polycarbonate membrane with a pore size of 0.4 µm, (AOX^®^ Nuclepore^®^ Polycarbonate Membrane, Whatman, United Kingdom). The release test was performed at a constant temperature of 32 ± 1 °C and a paddle rotation speed of 50 rpm. The acceptor medium was 500 mL ethyl alcohol 60% *w/w* or an aqueous solution of polysorbate 20 5% *w/w*. The design of the in vitro release experiment considering two investigated variables (the membrane and the acceptor medium) is presented in Figure 2. 

Therefore, for each hydrogel, four experiments were performed that differed from each other by the used membrane and/or acceptor medium. During the each experiment, which lasted a total of 240 min, 3 mL of the medium was withdrawn at specified intervals (after 15, 30, 60, 90, 120, 180, and 240 min), and the concentration of α-tocopherol was determined at the wavelength of maximum absorption (291 nm in ethyl alcohol 60% *w/w* and 294 nm in polysorbate 20 solution 5% *w/w*), using an Evolution 300 UV spectrophotometer (Thermo Fisher Scientific, Waltham, MA, USA). Based on the measured absorbance (A), the concentration of released α-tocopherol (c in mg/mL) was calculated. The equation of the calibration curve in the acceptor medium, ethyl alcohol 60% *w/w*, was as follows:

A = 5.9535c − 0.0027(4)

The calibration curve in the acceptor medium, polysorbate 20 5% *w/w*, is represented by Equation (5):

A = 7.7306c + 0.0017(5)

The cumulative amount of released α-tocopherol was expressed as a percentage in relation to the amount in the analyzed sample. Each hydrogel was analyzed in triplicate. The obtained profiles of the cumulative amount of α-tocopherol released as a function of time were compared with a model-independent approach. The Excel Add-In DDSolver was used to calculate the difference (*f*_1_) and similarity (*f*_2_) factors from the experimental data according to the following equations:

*f*_1_ = Σ_(t=1−*n*)_ |*R_t_* − *T_t_*|/Σ_(t=1−*n*)_ *R_t_*·100 (6)

*f*_2_ = 50log{[1 + 1/*n*·Σ_(t=1−*n*)_ (*R_t_* − *T_t_*)^2^]^−0.5^·100} (7)

where *n* is the number of samples, *R_t_* is the amount of α-tocopherol released (%) after time *t* (reference/sample 1), and *T_t_* is the amount of α-tocopherol released (%) after time *t* (test/sample 2). The release profiles of α-tocopherol were found to be similar for 0 < *f*_1_ < 15 and/or 50 < *f*_2_ < 100 [62,63].

## 3. Results

### 3.1. Influence of the Composition of the Microcapsule Wall on the Characteristics of α-Tocopherol-Loaded Microcapsules

In addition to the microcapsules prepared from LMWC/SLES in a mass ratio of 2:1, with or without the addition of a crosslinker (glutaraldehyde or formaldehyde), the crosslinker-free microcapsules with LMWC/SLES in a mass ratio of 1:1 were also prepared de novo, using the same procedure and under the same conditions. Table 1 summarizes the differences in the composition of the microcapsule wall and compares the results of the characterization of the microcapsules.

The moisture content and mean diameter of the crosslinker-free microcapsules and the microcapsules crosslinked with the aldehydes were similar, while the EE of α-tocopherol was lower in the crosslinker-free microcapsules (~70% m/m) than in the microcapsules with the chemically crosslinked walls (>80% m/m). A comparison of the results of the characterization of microcapsules without crosslinkers showed that changing the mass ratio of LMWC and SLES (from 2:1 to 1:1) for the wall structure of the microcapsules did not significantly affect the values of moisture content, mean diameter, and EE (Table 1). 

### 3.2. Compatibility of Microencapsulated α-Tocopherol with Carbomer Hydrogel 

The prepared blank hydrogel was a transparent and homogeneous soft semisolid with the scent of isopropyl alcohol. The recommended concentration of the carbomer Carbopol Ultrez^®^ 10 in hydrogels is 0.2–1.0% *w/w* [55]. In this work, all hydrogels were prepared with Carbopol Ultrez^®^ 10 at a concentration of 0.5% *w/w*, as it was recently reported that the microgel network is more sensitive to changes in the microenvironment caused by incorporation of liposomes at this carbomer concentration, while these were not observed at higher carbomer content (1%) [64]. The soft consistency of the blank hydrogel, which contained 25% isopropyl alcohol, was consistent with the known fact that carbomer hydrogels with a relatively high content of ethyl alcohol are softer than those that do not contain alcohol or contain it in low concentration [55]. Table 1 shows the names of the prepared microcapsule-loaded hydrogels and the calculated content of α-tocopherol in each hydrogel. The α-tocopherol content was calculated from the determined EE (Table 1), the microcapsule concentration in the hydrogel (5% *w/w*), and the α-tocopherol content in the microcapsule core (10% *w/w*), using Equation (8):

Content of α-tocopherol in hydrogel = EE/200 (8)

The concentration of microcapsules in all hydrogels was 5% *w/w* in order to exclude the influence of the content of dispersed microcapsules on the rheological properties of the microcapsule-loaded hydrogels. The calculated concentration of microencapsulated α-tocopherol in the investigated hydrogels was between 0.36 and 0.50% *w/w*, which was within the usual range for dermatological applications (0.1–1%) [11]. The incorporation of microcapsules containing α-tocopherol into the hydrogel vehicle resulted in a change in appearance. All six hydrogels with microencapsulated α-tocopherol were also homogeneous semisolids with a typical odor of isopropyl alcohol, but they were opalescent and softer than the blank hydrogel. The observed change in the optical properties (i.e., decrease in transparency) of the hydrogels is a consequence of the dispersion of the solid microcapsules in the hydrogel vehicle. In addition, poorly water-soluble microcapsule components, such as α-tocopherol, can migrate from the microcapsules into the aqueous phase of the carbomer hydrogel and reduce its transparency. The appearance of the hydrogels with microencapsulated α-tocopherol (W11, W21, G11, G12, F11, and F12) was tested after 1 and 2 months of storage in aluminum tubes at 20 ± 5 °C. The USP states that a change in color or noticeable changes in consistency and odor is the primary indicator of instability of semisolids [65]. No changes in color, homogeneity, consistency, and odor of the α-tocopherol microcapsule-loaded hydrogels were observed. The absence of syneresis, a time-dependent process manifested by the spontaneous expulsion of liquid from the gel as a result of changes in the gel structure due to insufficient concentration of a gelling agent, confirmed that the carbomer concentration was sufficient for the stabilization of the microcapsule-loaded hydrogels studied [66]. Carbomer hydrogels are susceptible to the development of microorganisms, especially when they contain a high concentration of water, and to ensure microbiological quality, they are usually preserved [67]. To ensure microbiological quality, freshly boiled purified water was used to prepare the hydrogels with microencapsulated α-tocopherol. In addition, the water phase contained 25% *w/w* isopropyl alcohol. It is already known that alcohols in a concentration of more than 15% *w/w* act as preservatives [65]. There were no visible signs of microbial growth, so the microbiological quality was not considered a critical quality attribute of the investigated hydrogels. Furthermore, the hydrogels were packaged in aluminum tubes, which protected them from evaporation and light during storage.

The results of measuring the pH of the blank hydrogel and the hydrogels with microencapsulated α-tocopherol after 48 h, 1 month, and 2 months of storage are summarized in Table 2.

During the preparation of the hydrogel vehicle, the carbomer molecules, which are tightly coiled together in the dry state of the polymer, partially uncoil during hydration and the deprotonated carboxyl groups lining the acrylic acid backbone become exposed. The resulting carbomer dispersion is acidic (pH ≈ 3) and of low viscosity, but neutralization of the carboxyl groups with triethanolamine resulted in negatively charged polymer chains that repel each other and continue to unfold, leading to an increase in the viscosity of the dispersion to form a transparent, colorless gel [68,69]. Triethanolamine is the recommended agent for neutralizing carbomers and for formulation stable hydrogels with a relatively high alcohol content [70]. The measured pH of the blank hydrogel 48 h after preparation was 7.22. The pH values of the freshly prepared hydrogels with microencapsulated α-tocopherol were in the range of 6.41–6.83 (Table 2). Dispersing the microcapsules into the hydrogel vehicle led to a decrease in pH by 0.39–0.81 pH units compared to the blank hydrogel. The observed decrease in pH could be a consequence of preparing the microcapsules from an acidic solution of LMWC (pH 4.0), which is a prerequisite for chitosan to be in the form of a polycation, as confirmed by a positive zeta potential [45]. It was also noted that the reduction in the pH of the hydrogel was greater for microcapsules without crosslinkers (and the higher proportion of acidified LMWC in the wall) than for microcapsules with crosslinkers (and the lower LMWC content; Table 2). Although a direct relationship between the content of LMWC in the wall of the microcapsule and the pH of hydrogels could not be established, based on the last observation, it was possible to hypothesize that the differences in the composition of the microcapsule wall had an impact on the pH of the hydrogels. Nevertheless, the observed pH decrease could not be considered significant to affect the compatibility between the microcarrier and the surrounding hydrogel, as all values fall within the pH range 6–10, where stable carbomer gels are formed [55]. The pH values of the blank hydrogel and all hydrogels with microencapsulated α-tocopherol also changed slightly over the course of two months and remained below the pH of the blank hydrogel (Table 2). Furthermore, the pH values of all hydrogels with microencapsulated α-tocopherol were close to the normally acidic pH of the skin (4–6), as well as the pH value recommended for cosmetic products for application on the skin (4.5–6.5) [71]. The hydrogels W11 and W21, which contained crosslinker-free microcapsules, had values that were closest to the physiological pH and can therefore be considered promising in terms of skin compatibility.

**Figure 3:**
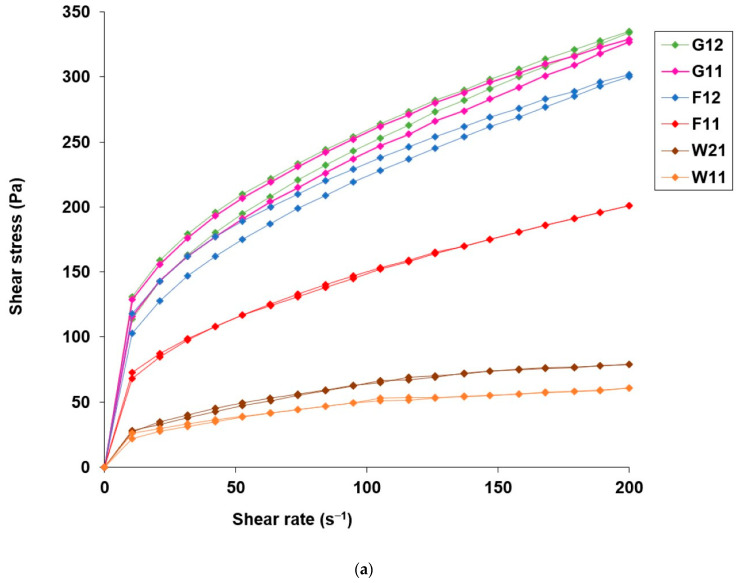

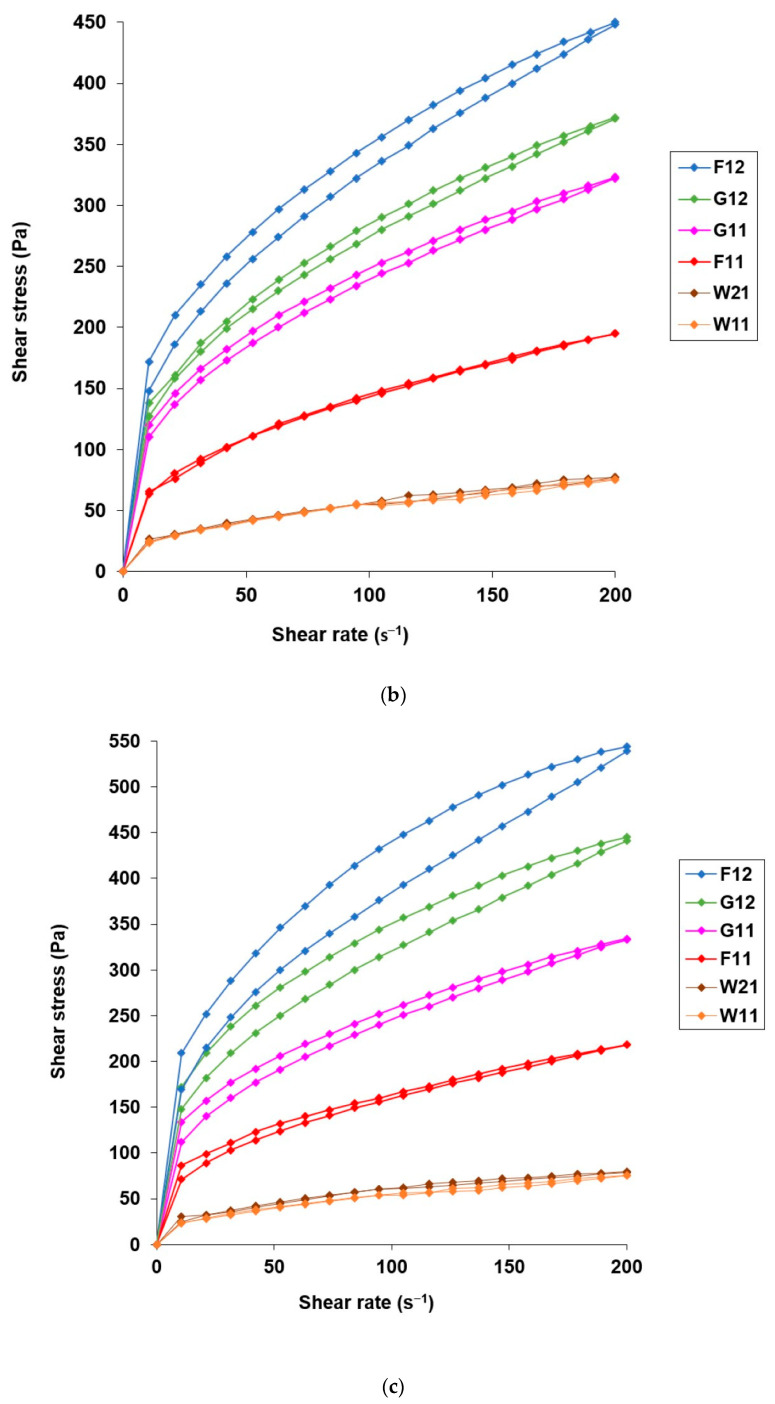
Flow curves of hydrogels with microencapsulated α-tocopherol after storage for: (**a**) 48 h, (**b**) 1 month, and (**c**) 2 months. The flow curve of the blank hydrogel was omitted from the figures to emphasize the subtle differences between the flow curves of the microencapsulated hydrogels within a relatively narrow range of shear stress.

The flow behavior of the blank hydrogel and the hydrogels with microencapsulated α-tocopherol, which were analyzed 48 h, 1 month, and 2 months after preparation, was pseudoplastic with thixotropy. The corresponding flow curves of the microcapsule-loaded hydrogels are shown in Figure 3. 

As shown in Figure 3a, dispersing microcapsules with α-tocopherol in the carbomer hydrogel did not lead to changes in the type of the flow, and the obtained pseudoplastic flow curves confirmed the homogeneity of all hydrogels. The apparent viscosity of all tested samples decreased with the increasing shear rate, which is typical for pseudoplastic systems and allows good spreadability on the skin, while the observed thixotropy (expressed as a hysteresis area) reflects a time-dependent shear-thinning property and reversibility of structural change, which is beneficial for the retention of the hydrogel at the application site [72]. The values of the rheological parameters, the maximum apparent viscosity (η_max_), the minimum apparent viscosity (η_min_), and the hysteresis area (H) are listed in Table 3.

The apparent viscosity and hysteresis area values of the blank hydrogel and the hydrogels with microencapsulated α-tocopherol were relatively low, which is typical for carbomer hydrogels with an aqueous phase containing cosolvents with lower polarity than water [73]. The hydrogels studied contain propylene glycol (10% *w/w*) and isopropyl alcohol (25% *w/w*), which have lower dielectric constants (32 and 17.9, respectively) than water (80.2), reducing the polarity of the aqueous phase. Reducing the polarity of the aqueous phase, reduces the attractive interactions between the polymer molecules and water, which limits the degree of hydration and solubility of the carbomer, thus reducing the viscosity and softening the consistency of the hydrogel [64]. In addition, the apparent viscosity of all hydrogels with microencapsulated α-tocopherol was lower than that of the blank hydrogel 48 h after preparation. This could be due to the disruption of the carbomer microgel when the microcapsules were introduced under agitation [53]. The mere presence of microcapsules also disturbs the coherence of the hydrogel network and reduces the apparent viscosity. Similar observations of the decrease in viscosity and thixotropy of hydrogels based on 0.5% carbomer caused by the incorporation of liposomes were reported by Dejeu et al. [64]. Finally, the observed decrease in the values of η_max_, η_min_, and H, in all hydrogels with microcapsules, could also be a consequence of the observed decrease in pH upon incorporation of microcapsules whose wall formed in an acidic environment as a prerequisite for the dissolution and ionization of LMWC and the formation of the complex with SLES. It is typical for carbomer hydrogels that the degree of crosslinking of the polymer chains and the apparent viscosity decrease with a decrease in pH, compared to neutral. In addition, the results of our study showed that differences in the composition of the microcapsule wall also affected the values of the rheological parameters investigated. At all time points, the hydrogels with microcapsules without crosslinkers (W21 and W11) showed lower values for apparent viscosity and hysteresis area compared to hydrogels with microcapsules crosslinked with glutaraldehyde or formaldehyde. The greatest reduction in apparent viscosity and thixotropy in hydrogels W11 and W21 may be related to the higher relative content of LMWC in the non-crosslinked microcapsule wall and the greater decrease in pH, which leads to greater local relaxation of the three-dimensional gel network in these hydrogels. At a lower pH, the degree of carbomer ionization and consolidation of the gel network based on repulsion of carbomer chains are limited. The aspect of potential interactions between carbomer and chitosan and their influence on the rheological properties of the investigated hydrogels was additionally considered. Although carbomers and chitosan have the properties of polyanions and polycations, respectively, a study by Gupta and Vyas [74] showed that an aqueous solution of carbomer (0.4% *w/v*) and chitosan (0.5% *w/v*) at pH 6 exhibited the properties of a low-viscosity liquid, as no significant interactions occurred between the amino groups of the chitosan and the carboxyl groups of the carbomer, while only at pH 7.4 was a significant increase in viscosity due to electrostatic interactions between the protonated amine groups of the chitosan and the carboxylate groups of the carbomer observed. Considering that the pH of the investigated hydrogels with microencapsulated α-tocopherol was below 7.4, where sol–gel conversion was observed, and that the microcapsule wall was chemically crosslinked in hydrogels F11, F12, G11, and G12, the contribution of potential interactions between LMWC from the microcapsule wall and carbomer to the apparent viscosity is probably negligible. 

**Figure 4:**
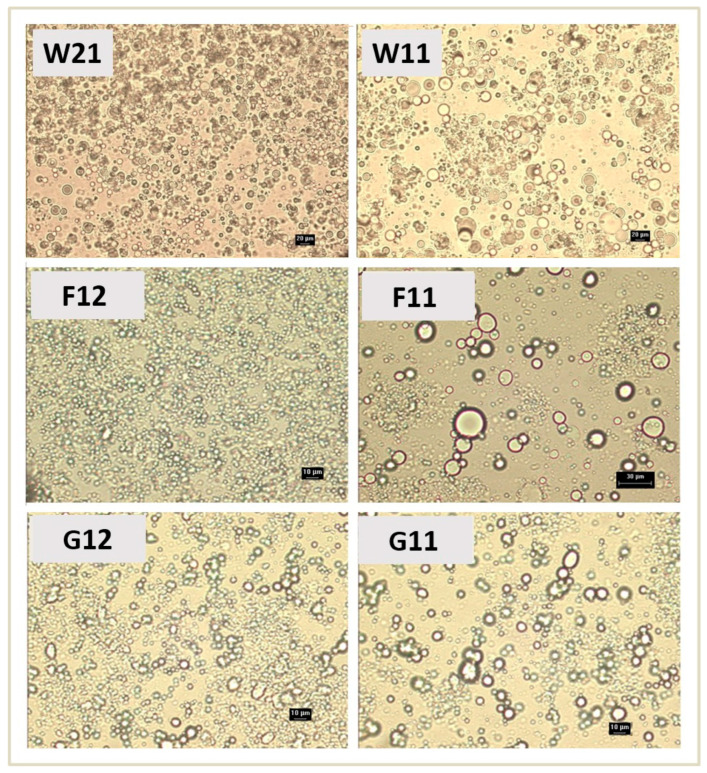
Photomicrographs of the hydrogels with microencapsulated α-tocopherol.

It was observed that, after 2 months the apparent viscosity and thixotropy of the microcapsule-loaded hydrogels increased (Table 3). The increase in apparent viscosity and thixotropy also occurred in the blank hydrogel, so it could be concluded that it is an inherent property of the carbomer hydrogel that occurs as a result of the time-dependent consolidation of the gel network. W21 and W11 showed the least changes in these rheological parameters as a function of time compared to the hydrogels containing microcapsules with crosslinkers (Table 3). The more extensive differences in the change in rheological parameters were observed for hydrogels F11 and F12. Therefore, it could be assumed that additional processes related to the wall composition (e.g., swelling) occur in the hydrogels with microencapsulated α-tocopherol, which could be reflected on the rheological properties. The results of the light microscopic examination of the hydrogels with microencapsulated α-tocopherol after 2 months of storage are shown in Figure 4. The swollen spherical microcapsules dispersed in the hydrogel vehicle were observed in all samples. The swelling of the microcapsules varied depending on the composition of the microcapsule wall. The size of the microcapsules without crosslinker was up to 20 μm in both hydrogels W11 and W21. The microcapsules prepared with glutaraldehyde as crosslinker had a size of up to 10 μm in the investigated hydrogels G11 and G12 at both investigated mass ratios. In contrast, the relative content of formaldehyde in the microcapsule wall significantly influenced the size of the microcapsules dispersed in the hydrogel vehicle. In hydrogel F12, the size of the microcapsules was up to 10 μm, while in hydrogel F11 it was up to 30 μm. Microscopic characterization of the dry microcapsules showed that their average size was less than 10 μm (Table 1). From the comparison of the sizes of the microcapsules in the dry state and in the hydrogels, it can be concluded that in all cases the incorporation of the microcapsules into the hydrogel led to an increase in the average size due to the swelling of the microcapsule wall. The size of the microcapsules with glutaraldehyde in the G11 and G12 hydrogels remained up to 10 μm (Figure 4), indicating the relatively small extent of swelling of the LMWC/SLES complex and the probably negligible influence of the relative content of this crosslinker and LMWC in the microcapsule wall. In the F11 hydrogel, the wall of the microcapsules contained formaldehyde in an equivalent mass ratio with LMWC, and the size increased several-fold compared to the dry state, so that some microcapsules reached a size of up to 30 μm, causing the overall heterogeneity of the system. The heterogeneity of microcapsule sizes in hydrogel F11 can be clearly seen in Figure 4. 

In the case of hydrogel F12, the microcapsule wall contains a lower proportion of LMWC compared to formaldehyde, and the swelling of the microcapsule wall was relatively uniform. These observations suggest that the intra- and intermolecular crosslinking of LMWC with aldehydes limits the swelling of the microcapsule wall to a greater extent at higher levels of both crosslinkers and in the presence of glutaraldehyde compared to formaldehyde. The swelling of the microcapsules in hydrogel F11 widened the size distribution of the microcapsule population present, causing the increase in polydispersity. It is well known that suspensions of predominantly smaller solid particles have a higher viscosity, while with increasing polydispersity the maximum packing density increases (i.e., the space can be filled more efficiently) and thus the apparent viscosity decreases for a given shear rate and solid fraction [75]. Accordingly, the polydispersity as a result of the inhomogeneous swelling of the dispersed microcapsules in hydrogel F11 could be an additional factor for the significantly lower apparent viscosity compared to other hydrogels with crosslinked microcapsule walls (F12, G11, and G12; Table 3) where the swelling did not significantly affect the homogeneity of the microstructure. Microcapsules without crosslinkers also swelled in the hydrogel vehicle (Figure 4). The polydispersity due to swelling was roughly estimated to be higher in hydrogel W11, which has a lower content of LMWC (and a higher content of SLES) in the microcapsule wall, compared to the microcapsules incorporated in hydrogel W21. The equivalent content of LMWC and SLES probably increases the wettability and swelling ability of the microcapsule wall in hydrogel W11. Therefore, the slightly lower apparent viscosity of hydrogel W11 compared to hydrogel W21 (Table 3) could also be related to the slightly higher polydispersity of the microcapsules in addition to the lower pH of hydrogel W11 (Table 2). Also, the overall higher polydispersity of the microcapsules in hydrogels W11 and W21 is probably an additional aspect that, in combination with the lower pH, reduces their apparent viscosity compared to the apparent viscosity of hydrogels with microcapsules with crosslinkers (F11, F12, G11, and G12).

The pseudoplastic flow behavior of the blank hydrogel and the hydrogels with microencapsulated α-tocopherol remained unchanged over 2 months. The unchanged flow behavior of the hydrogels indicated that despite the observed relaxation of the carbomer gel network in the presence of the microcapsules, its inherent stability was not affected. For each hydrogel containing microencapsulated α-tocopherol, the apparent viscosity values changed slightly during the indicated storage period, and a gradual increase in hysteresis area was observed after 1 month and 2 months, compared to the initial values measured 48 h after preparation (Table 3). Considering that the increase in hysteresis area was also detected for the blank hydrogel (Table 3) and that no significant change in the pH values occurred during the storage of all hydrogels compared to the initial values (Table 2), the observed increase in hysteresis area can only be interpreted as a consequence of the time-dependent consolidation of hydrogen bonds in the chains of carbomer during the storage of hydrogels [34].

### 3.3. Application Attributes of Hydrogels with Microencapsulated α-Tocopherol

The results of the evaluation of the spreading diameter (Φ) and the dispersion rate (t) as application-related attributes of the hydrogels with microencapsulated α-tocopherol are shown in Table 4.

All hydrogels with microcapsules had a slightly higher spreading diameter (Φ) and a slightly higher dispersion rate (t) in artificial sweat than the blank hydrogel, which could be due to the lower viscosity in the presence of the microcapsules. The spreading diameter of the microcapsule-loaded hydrogels was higher than the spreadability of the blank hydrogel (Table 4). Although it was not possible to establish a direct correlation between the investigated application properties and the maximum apparent viscosity, the spreading diameter was larger for each pair of microcapsule-loaded hydrogels with the same qualitative wall composition, when the apparent viscosity of the hydrogel was lower. Therefore, better spreading was observed in hydrogels W11, F11, and G11 compared to W21, F12, and G12 (Table 4). The appearance of the dispersions was visually inspected after 1, 2, and 5 min. The time required to completely disperse the sample in the artificial sweat so that the non-dispersed parts of the sample were not visible was recorded. The results were classified in the indicated intervals: “less than 1 min”, “more than 1 min and less than 2 min”, “more than 2 min and less than 5 min”, and “more than 5 min”. The average time for dispersion of all hydrogels with microcapsules was less than 5 min, while it was 6.5 min for the blank hydrogel. The hydrogels with microcapsules without crosslinkers (W11 and W21) disperse faster (less than 2 min) than the hydrogels with the microcapsules containing the crosslinkers. The largest spreading diameter and dispersion rate of less than 1 min were observed for hydrogel W11, making it particularly promising for topical application.

### 3.4. In Vitro Release of α-Tocopherol 

**Figure 5:**
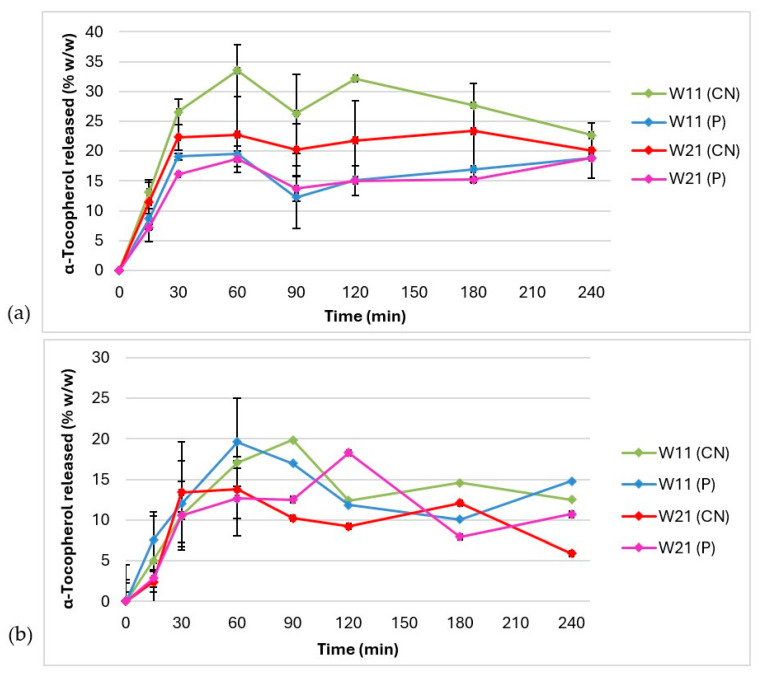
Release profiles of α-tocopherol from hydrogels W11 and W21 by diffusion through the membrane of cellulose nitrate (CN) or polycarbonate (P) in acceptor medium: (**a**) ethyl alcohol 60% *w/w* and (**b**) polysorbate 20 5% *w/w*.

The in vitro release kinetics of the microencapsulated α-tocopherol from hydrogels W11 and W21 were investigated. The α-tocopherol release profiles obtained are shown in Figure 5.

Figure 5a shows the release profiles of α-tocopherol during 240 min, when ethyl alcohol 60% *w/w* was used as the acceptor medium. It was observed that the maximum amount of α-tocopherol was released from both hydrogels after 60 min. When a cellulose nitrate membrane was used, a maximum of 33.51 ± 4.39% α-tocopherol was released from W11 and 22.77 ± 6.31% from W21, while the values in the presence of a polycarbonate membrane were lower and similar to each other (19.50 ± 1.33% (W11) and 18.69 ± 1.28 (W21)). In the profiles obtained, no consistent increase in the content of the released substance in the acceptor medium was observed between the 60th and 240th minute. It was assumed that, from the 60th minute, a balance was established in the acceptor medium between the process of release of α-tocopherol from the hydrogel, its dissolution in the acceptor medium, and the process of chemical degradation of dissolved α-tocopherol under the influence of daylight and air oxidation. Previously published results of tests on the release of α-tocopherol from the same microcapsules dispersed in ethyl alcohol 80% *v/v* showed that almost the entire amount of the active ingredient was released in 15 min by diffusion [45]. Since the microscopic analysis showed that the microcapsule wall was preserved in the hydrogels, it was assumed that the predominant release mechanism for α-tocopherol was also diffusion through the microcapsule wall. However, looking at the release profile of α-tocopherol from the hydrogels, it can be concluded that the incorporation of microcapsules into a carbomer hydrogel slowed down the release of α-tocopherol. This assumption was consistent with the observation reported by Hui et al. [76] that the diffusion-driven release of small molecules can be delayed by both encapsulation in a larger structure (e.g., liposomes or particles) and by suspension in the gel. However, the investigated carbomer hydrogel did not delay the release of α-tocopherol from the hydrogels through the membrane into the acceptor medium (i.e., no lag phase was observed in the release profiles). Therefore, we hypothesized that the release process was the result of a series of diffusion-based equilibrium processes, including partitioning between (i) the microcapsules and the surrounding hydrogel, (ii) the hydrogel and the membrane, and (iii) the membrane and the acceptor medium. The hypothesized model was supported by the observations of Gabbanini et al. [77] that the release of α-tocopherol from the aqueous phase of a water-in-oil emulsion was due to preferential diffusion across the membrane into the aqueous acceptor phase. We observed that both membranes affected the rate of diffusion of α-tocopherol from the hydrogel into the acceptor medium to different extents, and in both cases, the maximum amount of α-tocopherol released from hydrogel W11 was higher than that from hydrogel W21. This can be explained by the lower maximum apparent viscosity of hydrogel W11, which slows down the diffusion of α-tocopherol released from the oil core to the membrane less compared to W21. To evaluate the statistical significance of the similarities and differences of the obtained profiles, a model-independent analysis was performed. The calculated difference factor (*f*_1_) and similarity factor (*f*_2_) for the α-tocopherol release profiles in ethyl alcohol 60% *w/w* are shown in Table 5.

Based on the values obtained for both factors (Table 5), it was found that all profiles differ significantly from each other when the conditions 0 < *f*_1_ < 15 and 50 < *f*_2_ < 100 are taken into account. However, considering the *f*_2_ value as the only criterion, the profiles of the W11 and W21 hydrogels can be considered similar when the same membrane was used (W11 (P) vs. W21 (P), *f*_2_ = 61.73; W11 (CN) vs. W21 (CN), *f*_2_ = 58.76). This supported the assumption that diffusion through the membrane is an important stage of the release process and that the rate of diffusion through the membrane depends on the properties of the membrane. The value of *f*_2_ indicated that the choice of membrane had a greater effect on the release profiles of α-tocopherol from hydrogel W11 (not similar) than from hydrogel W21 (similar). In addition, the significant difference in the release profile of α-tocopherol from the W21 hydrogel in the presence of the polycarbonate membrane and from the W11 hydrogel in the presence of the cellulose nitrate membrane was determined using the *f*_2_ value. In this case, it could be assumed that the differences in the profiles are due to the combined effect of the viscosity of the hydrogel and the limiting effect of the membrane on the diffusion of α-tocopherol. In the case of hydrogel W11, the low apparent viscosity and higher permeability of the cellulose nitrate membrane likely enabled a significantly faster release of α-tocopherol compared to the more viscous hydrogel W21 in the apparatus with a less permeable polycarbonate membrane. These results suggest that the cellulose nitrate membrane could provide a more convenient in vitro model for studying the intrinsic release properties of different hydrogel formulations containing microencapsulated α-tocopherol. The choice of membrane was of greater importance in W11, as the release was less controlled by the viscosity of the hydrogel matrix, while the released α-tocopherol dissolved relatively quickly in the acceptor medium (ethyl alcohol 60% *w/w*). We also found that the cellulose nitrate membrane controlled the release of α-tocopherol to a lesser extent, so that it can be considered the better choice in combination with the acceptor medium, ethyl alcohol 60% *w/w*. 

Figure 5b shows the release profiles of α-tocopherol during 240 min, when an aqueous solution of polysorbate 20 5% *w/w* was used as the acceptor medium. When the cellulose nitrate membrane was used, a maximum of 19.86 ± 0.73% of α-tocopherol was released from W11 after 90 min and 13.78 ± 6.22% from W21 after 60 min. In the apparatus with a polycarbonate membrane, the maximum amounts of α-tocopherol released after 60 min were also very similar (19.60 ± 5.31% (W11) and 12.68 ± 0.46 (W21)). The maximum release amount of α-tocopherol from the hydrogel W11 in this acceptor medium was higher after 60 min and after 90 min than the release from W21 at the same time points with both membranes. The α-tocopherol release profiles in polysorbate 20 5% *w/w* between the 60th and 240th minute showed significant fluctuations. It was assumed that, in this interval, the processes of micellar solubilization of the released α-tocopherol and possible chemical degradation take place in this acceptor medium, although the dynamics of the processes of α-tocopherol release, solubilization, and chemical degradation are probably less coordinated than the dynamics of release, dissolution, and chemical degradation of α-tocopherol in ethyl alcohol 60% *w/w*. Considering that the maximum amounts of α-tocopherol released in polysorbate 20 5% *w/w* were similar or lower than those in ethyl alcohol 60% *w/w*, at the same time point, it could be concluded that the release rate of α-tocopherol was also influenced by the choice of acceptor medium and that ethyl alcohol 60% *w/w* was more suitable for this test. As already assumed, the reason for this is probably the different mechanism of dissolution of α-tocopherol in the acceptor media. Ethyl alcohol lowers the dielectric constant and polarity of water and thus provides a much more efficient and faster dissolution of lipophilic α-tocopherol compared to the micellar solubilization mechanism in the acceptor medium with polysorbate 20. This consideration is consistent with the data that show that the use of cosolvents can significantly increase the solubility of poorly soluble substances in water, compared to micellar solubilization [78]. Pinto et al. [23] also proposed water/ethanol 1:1 *v/v* as an acceptor medium in an in vitro study on the release of α-tocopherol from nanostructured lipid carriers (NLCs) for cosmetic purposes.

A model-independent analysis of the release profiles of α-tocopherol in polysorbate 20 5% *w/w* showed that they differed when both parameters (*f*_1_ and *f*_2_) were considered; however, when only *f*_2_ was considered, the difference between the profiles was not statistically significant (Table 5). This could suggest that, in addition to hydrogel viscosity and membrane permeability, the composition of the acceptor medium could also be an important factor influencing the release rate of α-tocopherol, although no statistically significant differences were observed between the profiles for polysorbate 20 5% *w/w* in contrast to ethyl alcohol 60% *w/w*. In order to question this assumption and eliminate the hydrogel apparent viscosity factor, the profiles of the individual hydrogels in different acceptor media and with different membranes were compared in a model-independent analysis. The values obtained for *f*_1_ and *f*_2_ are listed in Table 6.

A statistically significant difference was found between the α-tocopherol release profiles from both hydrogels (W11 or W21) in different media when the test was performed with a cellulose nitrate membrane. Also, the criteria for the similarity of the profiles (50 < *f*_2_ < 100 and 0 < *f*_1_ < 15) of the same hydrogel in different media were not met, even when a polycarbonate membrane was used. However, when only the similarity factor, *f*_2_, was considered, the values obtained for this parameter showed no statistically significant difference in the release profiles obtained in different media in the presence of a polycarbonate membrane (Table 6). In this way, the simultaneous influence of both the membrane and the composition of the acceptor medium on the α-tocopherol release profile from the tested hydrogels was demonstrated. 

## 4. Discussion

The microencapsulation of α-tocopherol as a chemically unstable active ingredient in topical formulations is an industrially feasible but underutilized technological strategy because formulation development involves numerous and complex challenges, including the choice of starting materials, compatibility of microcapsules and vehicles, and bioperformance of the formulation. The current regulations for cosmetic and pharmaceutical products limit the choice of excipients that can be used to form the wall of microcapsules. The encapsulation of α-tocopherol in the oily core of microcapsules with a wall based on the biopolysaccharide chitosan is promising for industrial use but usually requires strengthening the polymer wall of the microcapsule via chemical cross-linking with aldehydes. The current regulatory requirements for safe cosmetics and biocompatible topical pharmaceutical products impose the abandonment of the use of formaldehyde and glutaraldehyde, which raises the consideration of producing chitosan-based microcapsules without aldehyde crosslinkers. The consequences of not using aldehyde crosslinkers on the integrity of microcapsules and their performance as α-tocopherol carriers have hardly been investigated. In this study, the feasibility of the microencapsulation of α-tocopherol via the complex coacervation of LMWC and SLES at different mass ratios (2:1 and 1:1) without aldehyde crosslinkers was demonstrated. The absence of aldehyde crosslinkers led to a decrease in the EE compared to the value of this parameter in microcapsules with crosslinkers (Table 1). In our previous study, it was observed that microcapsules with aldehyde crosslinkers exhibited a high EE and rapid in vitro release of α-tocopherol in ethyl alcohol 80% *w/w* probably because a significant amount of α-tocopherol was also deposited on the surface of the microcapsules during the spray-drying process, whereas the crosslinker-free wall of microcapsules based on the LMWC/SLES 2:1 complex was likely more stable and better controlled the release of α-tocopherol [45]. Changing the mass ratio of LMWC/SLES to 1:1 in the present study did not result in significant differences in the moisture content, dry microparticle size, and EE, which may indicate that, at this mass ratio, the wall without a crosslinker also has sufficient strength to ensure the integrity of the microcapsules during spray drying, preventing the deposition of α-tocopherol on its surface. 

A further investigation was carried out with the aim of clarifying the influence of the exclusion of harmful glutaraldehyde and formaldehyde crosslinkers from the microcapsule wall and the significance of the LMWC/SLES mass ratio in the crosslinker-free microcapsule wall on their compatibility with the carbomer hydrogel and the application properties of the microcapsule-loaded hydrogels. All of the prepared microcapsule-loaded hydrogels were homogeneous, soft semisolids. The additional softening of the hydrogel consistency in the presence of microcapsules compared to the blank carbomer hydrogel can be considered an advantage, as it may facilitate the application on the skin, even on sensitive skin, which could be extremely important from the consumer’s point of view. At the same time, a change in the consistency of the system may be an indicator of a disturbance of the structure and its stability [65]. Therefore, the physicochemical characterization performed was necessary to assess the potential risk of compromising the stability of the hydrogels in the presence of microcapsules. The results of the pH measurement showed that the value of this parameter in the presence of microcapsules was slightly lower than that of the blank hydrogel (Table 2), but not below the lower limit of the range (pH 6) at which a critical suppression of the ionization of the carboxyl groups of the carbomer would be caused, thus limiting the repulsion of the gelling-agent molecules and the collapse of the gel network. Although lowering the pH of carbomer hydrogels should be considered as a factor that may compromise physical stability, bringing the pH of the topical formulation closer to the slightly acidic pH of the skin also increases its biocompatibility [71]. For hydrogels containing crosslinker-free microcapsules (W11 and W21), the presence of microcapsules led to a greater decrease in pH than for hydrogels containing microcapsules with crosslinkers (Table 2), making them preferrable in terms of compatibility with the physiological pH at the application site. Therefore, it can also be assumed that microcapsules without crosslinkers are more suitable carriers to reconcile the conflicting pH requirements in terms of the carbomer hydrogel stability and the biocompatibility of such a formulation.

The incorporation of microcapsules into the carbomer hydrogel did not lead to a change in the pseudoplastic flow behavior (Figure 3), which was also observed with the blank hydrogel, but reduced the apparent viscosity and hysteresis area compared to the blank hydrogel (Table 3). The decrease in the values of these rheological parameters could be due to (i) the disruption of the gel network structure during the incorporation of the microcapsules by stirring, (ii) the physical presence of microcapsules within the carbomer hydrogel [64], and (iii) a decrease in the pH of the hydrogel in the presence of microcapsules (Table 2). Since all hydrogels were prepared in the same way, at the same stirring speed, and contained the same concentration (5% *w/w*) of microparticles, which were very similar in size in a dry state (Table 1), these factors contributed equally to the change in the rheological parameters studied in each hydrogel. Therefore, the observed differences in apparent viscosity and hysteresis area values between the hydrogels could be attributed mainly to the different composition of the microcapsule wall and pH. This observation served as evidence to suggest that microcapsules with different wall compositions (i.e., the content of LMWC in the complex with SLES with or without an aldehyde crosslinker) caused a different decrease in pH, which affected the relaxation of the carbomer gel network to a different extent compared with the blank hydrogel. Microcapsules without crosslinkers caused the greatest reduction in apparent viscosity and hysteresis area, making hydrogels W11 and W21 more promising in terms of ease of topical application compared to the investigated hydrogels with microcapsules with aldehyde crosslinkers. Due to the lower apparent viscosity and thixotropy of W11 and W12, one might have expected them to be more suitable for easy application to the skin with better spreadability at the application site. The evaluation of the spreading diameter (Φ) and dispersion rate (t) in artificial sweat as application-related attributes of the hydrogels with microencapsulated α-tocopherol showed that they all had better application properties compared to the blank hydrogel. However, it is important to emphasize that the influence of the presence/absence of aldehyde crosslinkers in the microcapsule wall on the suitability of the hydrogels for topical application could not be evaluated from the Φ values obtained. Although this test was probably not accurate enough, the results obtained could still indicate differences in spreading between the tested hydrogels with microcapsules of the same qualitative wall composition and different apparent viscosity (W21 vs. W11; F12 vs. F11; G12 vs. G11). Dispersing hydrogels in artificial sweat proved to be a more reliable test to determine differences in the application properties of hydrogels with microcapsules, both with aldehyde crosslinkers (dispersion rate 2–5 min) and without them (dispersion rate less than 2 min). It was particularly interesting that a difference in the dispersion rate between the hydrogels W11 (less than 1 min) and W21 (1–2 min) could be determined in this test. The investigation of the application properties showed the superiority of hydrogel W11 compared to other tested hydrogels with microencapsulated tocopherol, including W21.

The storage of hydrogel samples with microencapsulated α-tocopherol in aluminum tubes for 2 months under controlled ambient conditions [58] did not result in changes in color, odor, homogeneity, consistency, pH (Table 2), and flow behavior (Table 3). Changes in apparent viscosity and hysteresis area were observed, with the values of these parameters being higher for all hydrogels after 2 months than those measured after 48 h of preparation, which can be explained by both the consolidation of the carbomer gel network that occurs over time [53,55] and the swelling of the polymer wall of the microcapsules in the aqueous phase of the hydrogel (Figure 4). Differences in the composition of the microcapsule wall caused differences in swelling. Hydrogels in which the swelling process disturbed the polydispersity of the microcapsules to a lesser extent showed less variation in apparent viscosity during the period studied and vice versa. Nevertheless, there was no evidence that the inherent stability of the hydrogels was compromised during a two-month storage period. The hydrogels W21 and W11 showed the least change in rheological parameters as a function of time compared to the hydrogels containing microcapsules with crosslinkers (Table 3), indicating a more favorable stability profile compared to the hydrogels containing microcapsules with aldehyde crosslinkers, especially formaldehyde.

The in vitro release of the active substance is central to understanding formulation performance at the site of application and is strongly recommended in the development of cosmetic products and topical pharmaceutical preparations [46,58]. The testing methodology and release mechanisms of microencapsulated active substances is an extremely challenging field of research for which there are no general guidelines and well-founded conclusions [79]. Particular challenges in testing the release of microencapsulated α-tocopherol in vitro are related to its liposolubility and inherent chemical instability, as well as the properties of the medium in which the microcapsules are dispersed. In this paper, a relatively simple method for the in vitro testing of the release of α-tocopherol from carbomer hydrogels with crosslinker-free microcapsules using compendial apparatus is proposed, focusing on the consideration of the influence of the composition of the microcapsule wall, the vehicle (carbomer hydrogel), the synthetic membrane (cellulose nitrate or polycarbonate), and the composition of the acceptor medium (ethyl alcohol 60% *w/w* or polysorbate 20 5% *w/w*). Based on the release profiles obtained, a release mechanism for α-tocopherol was proposed based on diffusion between the oil core of the microcapsule into the surrounding hydrogel matrix, from the hydrogel matrix into the synthetic membrane and from the synthetic membrane into the acceptor medium. During the first 60 min, the cumulative amount of α-tocopherol released increases through both membranes and in both acceptor media, indicating that a diffusion equilibrium has been established with a preferential release of α-tocopherol from both hydrogels (W11 and W21; Figure 5). However, after the 60th minute, fluctuations in the release profiles occurred (Figure 5), which are most likely a consequence of the chemical degradation of α-tocopherol in the acceptor medium under the influence of oxygen and daylight. To improve the quality of the data obtained, it can be suggested to perform the experiment in such a way that the acceptor medium is protected from daylight and a suitable antioxidant is added. The model-independent analysis of the release profiles (Table 5 and Table 6) indicated a simultaneous influence of the membrane type and the composition of the acceptor medium on the release profile of tocopherol. The importance of these experimental conditions was greater in the hydrogel that had a slightly higher apparent viscosity (W21), which probably slowed down the release to a greater extent. In both tested hydrogels, the model-independent comparison of the release profiles of α-tocopherol obtained in the presence of a polycarbonate membrane and the acceptor medium polysorbate 20 5% *w/w* yielded unclear conclusions, implying that the data obtained under the indicated conditions are not of sufficient quality for a model-independent statistical analysis. On the other hand, better data quality was obtained when the apparatus was used with a cellulose nitrate membrane and ethyl alcohol 60% *w/w* as the acceptor medium. Under later experimental conditions, significant differences were observed between the release profiles of α-tocopherol from W11 and W21 hydrogels.

## 5. Conclusions

Hydrogels with microencapsulated α-tocopherol, both with and without aldehyde crosslinkers, were soft pseudoplastic semisolids. The incorporation of microencapsulated α-tocopherol into a carbomer hydrogel resulted in the swelling of the microcapsule wall, a slight decrease in pH, and a decrease in apparent viscosity, as well as an improvement in the spreadability and dispersibility in artificial sweat compared to blank hydrogel. The unchanged appearance and flow behavior of the hydrogels during a two-month storage under controlled ambient conditions indicate that their inherent stability was not affected despite the observed changes within the carbomer gel network containing the microencapsulated α-tocopherol. The hydrogels with crosslinker-free microcapsules (W11 and W21) have potentially better properties for dermal application (pH, η_max_, η_min_, H, Φ, and *t*) than those with microcapsules containing the aldehyde crosslinkers. Although varying the amount of LMWC and SLES (2:1 or 1:1) in the wall of the microcapsules without crosslinkers had no significant effect on the moisture content, dry microcapsule size, and EE, the hydrogel W11 containing microcapsules with a mass ratio of 1:1 LMWC/SLES had a more favorable pH, lower apparent viscosity, better spreadability on a flat surface, and higher dispersion rate in artificial sweat compared to W21. The release of α-tocopherol from the oily core of the microcapsules was based on diffusion through the wall of the microcapsules and the surrounding carbomer hydrogel to the synthetic membrane and then through the membrane into the acceptor medium. A model-independent analysis of the release profiles revealed that the apparent viscosity of the hydrogel, the type of membrane, and the composition of the acceptor medium simultaneously influence the diffusion of α-tocopherol. A better quality of in vitro release data to elucidate the influence of the microcapsule wall composition on the release of α-tocopherol was obtained in an apparatus with a cellulose nitrate membrane and an acceptor medium in which the solubility of α-tocopherol is better (ethyl alcohol 60% *w/w*), so that these experimental conditions can be preferred when evaluating the quality of similar formulations.

## Figures and Tables

**Table 1:** The composition of the microcapsules, the parameters of the α-tocopherol-loaded microcapsules (moisture content, mean diameter, and EE), the labels of the hydrogels with microencapsulated α-tocopherol, and the calculated content of α-tocopherol in hydrogel.

Composition of the Microcapsule Wall	Moisture Content ± S.D. (% *w/w*)	Mean Diameter ± S.D. (µm)	EE ± S.D. (% *w/w*)	Hydrogel Label	Content of α-Tocopherol in Hydrogel (% *w/w*)
LMWC/SLES (mass ratio 2:1) without crosslinker	1.27 ± 0.081 ^1^	5.32 ± 0.252 ^1^	73.17 ± 0.64 ^1^	W21	0.37
LMWC/SLES (mass ratio 1:1) without crosslinker	1.35 ± 0.180	4.86 ± 0.29	71.12 ± 0.98	W11	0.36
LMWC/SLES (mass ratio 2:1) crosslinked with glutaraldehyde (LMWC/SLES-to-glutaraldehyde mass ratio 1:2)	1.30 ± 0.173 ^1^	5.84 ± 0.256 ^1^	99.50 ± 2.27 ^1^	G12	0.50
LMWC/SLES (mass ratio 2:1) crosslinked with glutaraldehyde (LMWC/SLES-to-glutaraldehyde mass ratio 1:1)	1.66 ± 0.376 ^1^	5.04 ± 0.331 ^1^	100.00 ± 3.55 ^1^	G11	0.50
LMWC/SLES (mass ratio 2:1) crosslinked with formaldehyde (LMWC/SLES-to-formaldehyde mass ratio 1:2)	1.76 ± 0.451 ^1^	6.21 ± 0.242 ^1^	82.30 ± 1.67 ^1^	F12	0.41
LMWC/SLES (mass ratio 2:1) crosslinked with formaldehyde (LMWC/SLES-to-formaldehyde mass ratio 1:1)	0.97 ± 0.312 ^1^	6.25 ± 0.224 ^1^	93.50 ± 1.08 ^1^	F11	0.47

^1^ Data previously published [45].

**Table 2:** pH of blank hydrogel and hydrogels with microencapsulated α-tocopherol.

	pH		
Sample	48 h	1 Month	2 Months
Blank hydrogel	7.22	7.17	7.20
W21	6.44	6.51	6.60
W11	6.41	6.48	6.53
F12	6.68	6.77	6.82
F11	6.60	6.63	6.71
G12	6.83	6.87	6.88
G11	6.58	6.64	6.68

**Table 3:** Maximum apparent viscosity (η_max_) at 10.5 s^−1^, minimum apparent viscosity (η_min_) at 200 s^−1^, and hysteresis area (H) of the hydrogels with microencapsulated α-tocopherol.

	48 h			1 Month			2 Months		
Sample	η_max_ (Pa∙s)	η_min_ (Pa∙s)	H (Pa/s)	η_max_ (Pa∙s)	η_min_(Pa∙s)	H (Pa/s)	η_max_ (Pa∙s)	η_min_ (Pa∙s)	H (Pa/s)
Blank hydrogel	16.4 ± 0.14	2.41 ± 0.01	2614.99	18.7 ± 0.32	4.41 ± 0.06	2934.09	20.5 ± 0.16	3.38 ± 0.07	3512.08
W21	2.54 ± 0.05	0.62 ± 0.05	7.51	2.25 ± 0.34	0.51 ± 0.09	6.36	2.71 ± 0.51	0.61 ± 0.02	8.33
W11	2.18 ± 0.06	0.54 ± 0.01	6.17	2.22 ± 0.07	0.54 ± 0.01	5.88	2.34 ± 0.14	0.49 ± 0.03	6.24
F12	11.2 ± 0.42	1.51 ± 0.02	1933.9	15.4 ± 0.37	2.15 ± 0.04	2985.64	19.8 ± 0.75	2.72 ± 0.06	8319.54
F11	6.9 ± 0.69	1.01 ± 0.03	8.11	6.2 ± 0.08	0.98 ± 0.02	91.88	8.12 ± 0.31	1.09 ± 0.02	1049.11
G12	12.45 ± 0.49	1.67 ± 0.01	2045.86	13.1 ± 0.65	1.86 ± 0.07	1552.81	16.3 ± 0.54	2.22 ± 0.07	4810.33
G11	12.15 ± 0.35	1.65 ± 0.02	2481.12	11.4 ± 0.29	1.61 ± 0.02	1533.52	12.7 ± 0.41	1.67 ± 0.03	2227.38

**Table 4:** Spreading diameter (Φ) and the dispersion rate (t) of blank hydrogel and hydrogels with microencapsulated α-tocopherol.

Sample	Φ (mm) ± S.D.	*t* (min)
Blank hydrogel	19.5 ± 0.5	>5
W21	30.7 ± 1.5	1–2
W11	43.3 ± 2.5	<1
F12	28.7 ± 2.5	2–5
F11	39.7 ± 1.2	2–5
G12	36.7 ± 0.6	2–5
G11	38.3 ± 1.5	2–5

**Table 5:** Values of difference factor (*f*_1_) and similarity factor (*f*_2_) of α-tocopherol release profile through cellulose nitrate (CN) and polycarbonate (P) membrane in different acceptor media.

Acceptor Medium	Ethyl Alcohol 60% *w/w*		Polysorbate 20 5% *w/w*	
Samples	*f*_1_	*f*_2_	*f*_1_	*f*_2_
W11 (CN) vs. W11 (P)	39.25	47.36	18.12	65.24
W21 (CN) vs. W21 (P)	26.29	61.73	32.72	66.90
W11 (CN) vs. W21 (CN)	21.92	58.76	33.39	64.87
W11 (P) vs. W21 (P)	26.29	61.73	32.54	66.03
W11 (CN) vs. W21 (P)	42.45	46.20	30.71	56.98
W21 (CN) vs. W11 (P)	22.19	64.41	52.82	57.74

**Table 6:** The influence of acceptor media and a membrane on difference factor (*f*_1_) and similarity factor (*f*_2_) of α-tocopherol release profiles from the hydrogels W11 and W21.

Membrane	Cellulose Nitrate		Polycarbonate	
Samples	*f*_1_	*f*_2_	*f*_1_	*f*_2_
W11 (ethyl alcohol 60% *w/w*) vs. W11 (polysorbate 20 5% *w/w*)	49.45	46.15	24.69	60.03
W21 (ethyl alcohol 60% *w/w*) vs. W21 (polysorbate 20 5% *w/w*)	52.82	48.99	34.08	58.11

## Data Availability

The original contributions presented in the study are included in the article, further inquiries can be directed to the corresponding author.
